# Intersection of Surging Firearm Sales and COVID-19, Psychological Distress, and Health Disparities in the US—A Call for Action

**DOI:** 10.1001/jamanetworkopen.2020.34017

**Published:** 2021-01-04

**Authors:** Katelin Hoskins, Rinad S. Beidas

**Affiliations:** Department of Psychiatry, Perelman School of Medicine, University of Pennsylvania, Philadelphia;; Department of Psychiatry, Perelman School of Medicine, University of Pennsylvania, Philadelphia; Penn Implementation Science Center at the Leonard Davis Institute of Health Economics, University of Pennsylvania, Philadelphia.

Mass shooting events (eg, Sandy Hook, San Bernardino) and national elections have historically been associated with increased firearm sales in the US.^1^ Against the backdrop of other salient phenomena that have occurred in 2020—the coronavirus disease 2019 (COVID-19) pandemic, rising psychological distress, police violence against minority groups, protests for racial justice, and highly polarized politics—the popular press has reported increased firearm acquisition. In a methodologically rigorous, cross-sectional population-representative, survey of 2870 adults in California, Kravitz-Wirtz and colleagues^2^ analyzed public concern about (1) violence against themselves and others, (2) the prevalence of and reasons for firearm and ammunition acquisition, and (3) firearm storage practices. Their findings demonstrate that concern about violence has increased robustly during the pandemic. Specifically, survey respondents expressed concern that someone they know may intentionally harm another person (12.2%) or themselves (13.1%) owing to experiencing pandemic-related losses. The authors estimated that approximately 110 000 Californians acquired firearms due to the pandemic, including approximately 47 000 new owners who primarily acquired them for self-protection. Nearly 1 in 5 respondents reported storing at least 1 firearm loaded and not locked up, with approximately 7% of these owners reporting this change in storage practice owing to the pandemic. Of owners who stored firearms in this way, half lived in households with children. These findings are highly pertinent with regard to 3 matters that require attention amid the current charged social and political context: (1) implications for safe storage initiatives broadly; (2) safe storage initiatives specific to suicide prevention amid rising psychological distress; and (3) the intersection of these matters within an inequitable society that is rife with health disparities for racial/ethnic minority communities.

Californians’ acquisition of firearms reflects a broader national trend. The increasing numbers of new firearm owners has implications for injury prevention research and practice, particularly primary prevention efforts via safe firearm storage initiatives. Reasons for ownership are a key focal point for understanding how to target safe storage programs. As Kravitz-Wirtz et al^2^ point out, owning firearms primarily for protection increases the likelihood of less safe storage practices, a substantial risk factor for firearm-related injuries and death (eg, unintentional shootings by children), generating a protection paradox. New owners who purchased a firearm in response to the pandemic along with individuals who reported changing their storage practices are critical constituencies to engage to promote a shared goal of preventing unauthorized access to their firearms and ultimately to prevent firearm injury and mortality. A harm reduction approach creates opportunities for engagement to increase the adoption of programs that may lower risk.^3^ For example, creative design of virtual firearm safety courses that maintain core components (ie, safe handling and storage) may better reach the new owners who have fewer training options amid COVID-19 mitigation measures (eg, temporary closure of indoor training ranges, limited course sizes). To better understand the association between firearm training and subsequent safety behaviors, research is needed that identifies the active components of training courses and assesses the utility of alternative delivery avenues (ie, online).

Increased firearm acquisition has occurred within the context of increasing psychological distress given the morbidity, mortality, and mitigation efforts of COVID-19, with an increase in established suicide risk factors (eg, personal loss, isolation) nationally.^4^ Economic recession and financial stress are also risk factors for suicide attempts and deaths, although no evidence yet exists for increased pandemic-related suicides.^5^ Targeting safe firearm storage programs within the context of suicide prevention efforts given the established association between firearm access and suicide is critical. Firearms are the most lethal method for suicide, and most firearm deaths are suicides.^6,7^ Storing firearms locked and unloaded can be effective in reducing the number of suicides, with data indicating that even a modest increase in safer storage could reduce firearm-related deaths in youth.^8^ This finding extends to adults; one study showed that firearm owners who kept their firearms locked or unloaded were 60% less likely to die from firearm-related suicide compared with owners who stored their firearms unlocked and/or loaded.^9^ Evidence-based interventions that create time and space between at-risk individuals and lethal means are critical, particularly within the context of increased firearm acquisition and increased psychological distress nationally.

In addition, this study amplifies the need to further expose and address the structural inequities in the US, illustrated by the inequitable burden of the COVID-19 pandemic and firearm-related violence in racial/ethnic minority communities. Kravitz-Wirtz and colleagues^2^ underscore how COVID-19 has aggravated inequities in the conditions (eg, poverty, unemployment) that intersect with community violence. Firearm violence, police violence, and COVID-19 deaths have taken a devastating toll on racial/ethnic minority communities. Furthermore, members of racial and ethnic minority groups have experienced disproportionately worse mental health outcomes, elevated suicidal ideation, and increased substance use associated with COVID-19.^4^ These findings engender additional concern when layered onto the prepandemic pattern of increasing suicide rates in young Black children^10^ and surging purchases of firearms by Black Americans in the first half of 2020. Crosscutting social crises signal a major reckoning with the structural institutions (eg, racist policies) that continue to harm the health and safety of racial/ethnic minority communities. As we work to remedy upstream factors, we must also conduct research in partnership with racial/ethnic minority communities to leverage priorities and strengths, target long-standing barriers to mental health care access and use, and adapt interventions in culturally meaningful ways that enhance equity.

The study by Kravitz-Wirtz et al^2^ provides insight into people’s concerns about violence, firearm purchasing, and storage practices in response to the COVID-19 pandemic in California. The findings may have important implications for firearm safe storage programs, suicide prevention amid rising psychological distress, and health equity. Given the surge in new ownership of firearms, we believe that collaboration with firearm owning constituencies is essential to address the potential downstream adverse effects of increases in firearm ownership with regard to injury and suicide prevention. Furthermore, the inequities in communities of racial/ethnic minority groups must be addressed. The influx of recent funding from the Centers of Disease Control and Prevention and the National Institutes of Health to fund firearm injury prevention research is a reason for optimism as researchers keep pushing to understand the best firearm-related practices to improve health equitably.
